# Regulation of androgen receptor expression by enhancer elements in prostate cancer

**DOI:** 10.1038/s12276-025-01624-9

**Published:** 2026-01-16

**Authors:** Sudeep Khadka, Hee-Young Jeon, Arif Hussain, Jianfei Qi

**Affiliations:** 1 Department of Biochemistry and Molecular Biology, University of Maryland, Baltimore, MD USA; 2 Marlene and Stewart Greenebaum Comprehensive Cancer Center, Baltimore, MD USA; 3 Baltimore VA Medical Center, Baltimore, MD USA

**Keywords:** Prostate cancer, Gene regulation

## Abstract

Androgen receptor (AR) overexpression is a key mechanism driving the development of castration-resistant prostate cancer (CRPC). This can result from multiple factors, including enhanced AR transcription and increased stability of AR mRNA and protein. In clinical CRPC samples, one cause of AR overexpression is gene amplification at the AR locus, which leads to elevated AR transcript and protein levels. In addition, increased activity or copy number of enhancer elements near the AR gene has been associated with elevated AR transcription. These regulatory regions interact with the AR gene promoter through enhancer–promoter looping, thereby enhancing AR mRNA transcription. Elucidating the role of these enhancer elements in driving AR overexpression and aberrant AR signaling may uncover new therapeutic targets for CRPC.

## Enhancer Elements Drive Androgen Receptor Overexpression in Prostate Cancer

Prostate cancer is a major health issue for men worldwide, with advanced stages being particularly deadly. The review focuses on understanding why some prostate cancers become resistant to current treatments. Researchers explored how certain parts of the DNA near the androgen receptor (AR) gene contribute to this resistance. The studies used various techniques to examine prostate cancer cells and tissues. They identified specific DNA regions, called enhancers, that boost AR gene transcription. These enhancers can become more active or increase in number, leading to higher AR levels, which helps cancer resist treatment. Key findings show that these enhancers, when amplified or activated, significantly increase AR expression. This contributes to treatment resistance in advanced prostate cancer. The researchers suggest that targeting these enhancers could offer new ways to treat resistant prostate cancer.

## Introduction

Prostate cancer (PCa) is the second most common cancer among men worldwide. Metastatic castration-resistant PCa (CRPC) is the leading cause of mortality in patients with PCa. Androgen deprivation therapy, which suppresses androgen receptor (AR) signaling, remains the first-line treatment for metastatic PCa. Although androgen deprivation therapy initially induces clinical remission, the disease almost invariably recurs within 2–3 years and progresses to the lethal CRPC stage. Current treatment options for metastatic CRPC include taxane-based chemotherapy (docetaxel or cabazitaxel) and second-generation AR pathway inhibitors (enzalutamide or abiraterone), which modestly extend survival. However, CRPC ultimately develops resistance to these therapies, often through reactivation of AR signaling.

A major driver of CRPC progression and resistance to AR-targeted therapy is the reactivation of AR transcriptional activity through multiple mechanisms, including AR overexpression, mutations, alternative splicing, upregulation of AR cofactors and intratumoral androgen biosynthesis^1^. AR splice variants such as AR-V7, a constitutively active isoform, is generated through cotranscriptional splicing of AR pre-mRNA. Enhanced transcription of the *AR* gene can therefore increase both full-length AR mRNA and AR splice variants, each contributing to reactivation of AR signaling during CRPC progression. Both AR mRNA and protein levels increase as resistance to anti-androgen therapy emerges. In xenograft models, elevated AR expression was shown to be both necessary and sufficient for CRPC development^2^, highlighting AR overexpression as a key adaptive feature of CRPC progression.

**Fig. 1:**
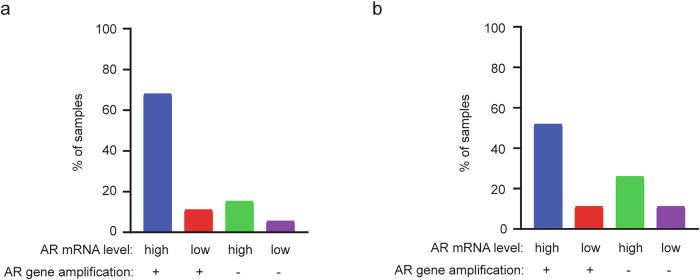
Correlation between AR gene amplification and AR mRNA expression in two CRPC datasets. **a** The percentages of CRPC samples classified into the four categories based on AR mRNA levels and AR gene amplification status in the SU2C/PCF Dream Team, *PNAS* 2019 dataset (*n* = 144). Data were obtained from cBioPortal. Only patients with both gene copy number variation and mRNA expression data were included in the analysis. **b**, The percentages of CRPC samples classified into four categories based on AR mRNA levels and AR gene amplification status in the MCTP, *Natur*e 2015 dataset (*n* = 27). Data were obtained from cBioPortal. Only patients with both gene copy number variation and mRNA expression data were included in the analysis.

AR gene amplification occurs in approximately 30–50% of CRPC cases and contributes to increased AR mRNA expression^3^. In addition to gene amplification, transcriptional upregulation also plays a role in AR mRNA overexpression in CRPC. In two CRPC datasets with matched measurements of AR gene copy number and AR mRNA levels, 15% (Fig. 1a) and 26% (Fig. 1b) of samples had high AR mRNA expression without AR gene amplification, suggesting that transcriptional upregulation independent of gene amplification contributes to AR overexpression. In total, 68% (Fig. 1a) and 52% (Fig. 1b) of samples showed high AR mRNA levels accompanied by AR gene amplification, which may result from gene amplification alone or from a combination of amplification and transcriptional activation. Supporting the latter, 11% of samples with AR gene amplification did not display elevated AR mRNA levels (Fig. 1a,b), indicating that amplification alone may be insufficient to drive AR overexpression in some CRPC cases. Together, these analyses highlight an important contribution of transcriptional mechanisms to AR mRNA upregulation in CRPC, both in the presence and in the absence of AR gene amplification.

**Fig. 2:**
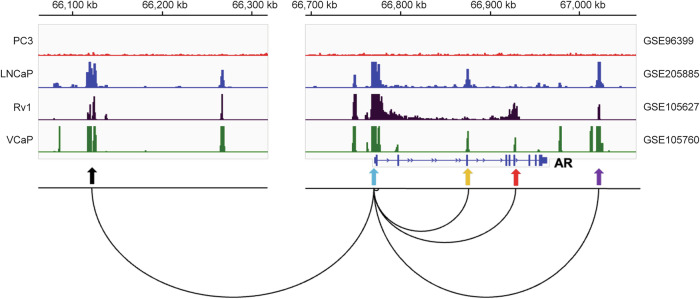
Representative visualization of enhancer–promoter interactions at the AR locus in human PCa cells. H3K27ac ChIP-seq peaks at the AR gene and its associated enhancer regions are shown. Publicly available H3K27ac ChIP-seq datasets from human PCa cells (AR-positive: LNCaP, Rv1 and VCaP; AR-negative: PC3) were downloaded from the Gene Expression Omnibus (GEO) database and visualized using Integrative Genomics Viewer (IGV) software. A representative chromatin loop illustrating the interaction between each AR enhancer and AR promoter is shown (adapted from Giambartolomei et al.^7^). The *cis*-regulatory elements are indicated by arrows: black (upstream enhancer), blue (promoter), yellow (intron 2 enhancer), red (intron 3 enhancer) and purple (downstream enhancer).

Several transcription factors have been reported to either promote or suppress AR gene transcription^4,5^, although their specific contributions to AR upregulation during CRPC progression remain poorly understood. Histone H3 lysine 27 acetylation (H3K27ac) is a well-established marker of active enhancers. Studies using chromatin immunoprecipitation (ChIP) followed by sequencing (ChIP-seq) have identified multiple H3K27ac peaks across the AR gene locus (Fig. 2), some of which function as enhancers that regulate AR transcription. In this mini-review, we highlight AR gene enhancers and their potential roles in driving AR mRNA upregulation in CRPC.

## AR upstream enhancer

Takeda et al. reported that CRPC tumors exhibited amplification of an enhancer region (~9 kb) located approximately 650 kb upstream of the AR gene transcription start stie^6^. We refer to this site as the AR upstream enhancer. Strong H3K27ac ChIP-seq signals were observed at the AR upstream enhancer region in CRPC samples (*n* = 4), but not in primary PCa samples (*n* = 6)^6^, suggesting CRPC-specific enhancer activation. Notably, the AR upstream enhancer region overlaps with three DNase I hypersensitive (DHS) sites previously identified in LNCaP cells and was shown to physically interact with the AR promoter in chromosome conformation capture (3C) analyses^6^. Consistently, high-throughput Chromosome Conformation Capture with ChIP (Hi-ChIP) data of H3K27ac in LNCaP cells demonstrated that the AR upstream enhancer forms a chromatin loop with the AR promoter^7^. To test its functional relevance, the authors performed a CRISPR interference (CRISPRi) screen using a pooled single guide RNA library targeting the upstream enhancer region in LNCaP cells. CRISPRi-mediated repression of the DHS peaks substantially reduced AR mRNA expression and cell proliferation, effects that were rescued by forced AR overexpression^6^. These findings demonstrate that the AR upstream enhancer functionally drives AR expression. Furthermore, CRISPR-mediated knock-in of a single copy of this upstream enhancer in LNCaP cells increased AR expression and conferred resistance to androgen deprivation and enzalutamide treatment^6^, highlighting its role in promoting castration resistance. However, the specific transcription factors that bind to this upstream enhancer and regulate AR transcription remain to be identified.

Amplification of the AR upstream enhancer is more frequent in CRPC than in primary PCa^3,8–10^. The copy number of this enhancer region is further increased in CRPC samples following treatment with AR pathway inhibitors such as enzalutamide or abiraterone^11^. In a cohort of 101 CRPC samples, 67% exhibited co-amplification of the AR gene and its upstream enhancer region, which was associated with elevated AR mRNA expression compared with samples lacking amplification at either locus (*n* = 18)^8^. Notably, 13% of CRPC samples showed amplification of the upstream enhancer alone, which also correlated with increased AR expression^8^, supporting the notion that enhancer amplification alone can drive AR overexpression. Samples displaying co-amplification of the AR gene and its upstream enhancer had higher AR expression levels^8^, suggesting an additive effect. Together, these findings indicate that amplification of the AR upstream enhancer, particularly when co-amplified with the AR gene, play a key role in driving AR overexpression in CRPC.

## AR distal promoter

EZH2 is an oncogene that is highly upregulated in various cancer types, including PCa^12–14^. As the catalytic subunit of the Polycomb Repressive Complex 2 (PRC2), EZH2 mediates gene silencing through trimethylation of histone H3 at lysine 27 (H3K27me3). However, EZH2 can also regulate gene expression independently of PRC2. For example, EZH2 has been reported to interact with AR and promote AR target gene expression independently of its canonical role in PRC2^15,16^.

EZH2 has been reported to promote AR mRNA expression^17^. Knockdown of EZH2 decreases AR mRNA levels in PCa cells, while EZH2 overexpression has the opposite effect. EZH2 ChIP-seq analysis and luciferase reporter assays revealed that EZH2 binds to a distal promoter region of the AR gene, located 1.7–2.5 kb downstream of the AR gene transcription start site, and enhances AR transcriptional activity in LNCaP cells^17^. Deletion of the EZH2-binding region using CRISPR–Cas9 abolished the effect of EZH2 knockdown on AR mRNA levels^17^, confirming that this site is functionally required for EZH2-mediated upregulation of AR transcription.

This distal promoter region lacks the repressive H3K27me3 mark and instead displays the active H3K27ac modification^17^. Importantly, knockdown of SUZ12, another core component of PRC2, did not affect AR mRNA levels^17^, suggesting that EZH2 functions independently of PRC2 in this context. Furthermore, a catalytically inactive EZH2 mutant retained the ability to regulate AR mRNA levels, indicating that EZH2’s methyltransferase activity is not required for regulation of AR gene expression. Similarly, EZH2 was reported to promote AR chromatin binding independent of PRC2 and H3K27me3^16^. Together, these findings suggest that EZH2 enhances both AR expression and chromatin binding in CRPC. Notably, EZH2 is also overexpressed in the AR-negative neuroendocrine PCa (NEPC) and functions as a driver for NEPC development^18–20^. In contrast to its noncanonical role in enhancing AR expression and activity in CRPC^16,17^, EZH2 in NEPC promotes H3K27me3 deposition at epithelial genes, including AR target genes, through canonical PRC2-mediated gene silencing^18,21^.

The precise mechanism by which EZH2 promotes AR transcription, particularly whether it facilitates recruitment of other transcription factors, remains to be elucidated. Interestingly, the RAR-related orphan receptor gamma (ROR-γ) was reported to directly bind to a site 2.3 kb downstream of the AR gene transcription start site, overlapping with the EZH2-binding region within the AR distal promoter^22^. The authors showed that ROR-γ is overexpressed in CRPC and promotes AR expression. ROR-γ knockdown or pharmacological inhibition reduces AR mRNA and protein levels in various PCa cell lines^22^. Notably, deletion of this ROR-γ binding site within the AR distal promoter using CRISPR–Cas9 resulted in reduced AR mRNA expression in C4-2 cells^22^. Furthermore, treatment with a ROR-γ antagonist decreased active histone marks (H3K4me3 and H3K27ac), as well as the binding of co-activators NCOA1, NCOA3 and RNA polymerase II at the ROR-γ binding site^22^. These findings support a model in which ROR-γ promotes AR transcription by binding to the distal AR promoter and recruiting co-activators and RNA polymerase II. It will be of interest to determine whether EZH2 cooperates with ROR-γ or its associated cofactors to drive AR expression at the AR distal promoter.

## AR intron 2 enhancer

AR can function as both a transcriptional activator and repressor of its target genes. Notably, AR has been reported to repress its own transcription in an androgen-dependent manner^23^. In VCaP cells, dihydrotestosterone (DHT) treatment led to a reduction in AR mRNA levels, an effect that was blocked by an AR antagonist, suggesting that DHT-bound AR represses AR gene expression. ChIP assays further revealed reduced RNA polymerase II binding at exon 1 of the AR gene upon DHT treatment. Subsequent ChIP assays demonstrated that DHT induces AR binding to an AR binding site (ARBS) located in intron 2 of the AR gene, accompanied by decreased levels of enhancer-associated histone marks H3K4me1 and H3K4me2 in both VCaP cells and LNCaP-CSS3 cells (LNCaP cells maintained in steroid-depleted medium for over 3 weeks). Moreover, AR ChIP followed by 3C analysis revealed a DHT-dependent interaction between AR, the intron 2 ARBS and the AR promoter. These findings suggest that the ARBS within intron 2 functions as an enhancer, and that DHT-liganded AR represses AR transcription by binding to this enhancer and disrupting its activity. We refer to this site as the AR intron 2 enhancer. Consistently, Hi-ChIP data of H3K27ac in LNCaP cells demonstrated that the AR intron 2 enhancer forms a chromatin loop with the AR promoter^7^.

Lysine-specific demethylase 1 (LSD1) is a histone demethylase that removes methyl groups from either the activating mark H3K4 or the repressive mark H3K9, thereby functioning as a transcriptional repressor or activator in a context-dependent manner. LSD1 has previously been shown to interact with AR and promote AR target gene expression by demethylating repressive H3K9 marks^24,25^. In this study, ChIP assays demonstrated that DHT treatment promoted the recruitment of LSD1 to the AR intron 2 enhancer in both VCaP and LNCaP-CSS3 cells^23^. Knockdown of LSD1 by siRNA or inhibition of its activity attenuated the DHT-induced suppression of AR expression, indicating that LSD1 is required for this repression. Notably, LSD1 knockdown did not affect DHT-induced AR binding to the intron 2 enhancer^23^, suggesting that LSD1 acts downstream of AR recruitment. These findings support a model in which DHT-liganded AR recruits LSD1 to the AR intron 2 enhancer to repress AR gene transcription. The mechanism by which LSD1 exerts this repressive effect remains to be determined.

In summary, DHT-liganded AR represses AR transcription by recruiting LSD1 to the intron 2 enhancer under physiological androgen levels. Under androgen deprivation condition, the loss of AR–LSD1 binding to this enhancer will remove the repressive effect of the AR–LSD1 complex, leading to increased AR gene transcription. This derepression probably contributes to the upregulation of AR mRNA observed in CRPC.

## AR intron 3 enhancer

SMAD3 is a key effector of canonical TGF-β signaling, in which activation of the TGF-β receptor type I (TGFBR1) leads to phosphorylation of SMAD2 and SMAD3. These phosphorylated SMADs then heterodimerize with SMAD4, forming complexes that translocate to the nucleus and regulate gene expression^26^. While SMAD4 functions as a tumor suppressor in PCa^27,28^, SMAD3 is overexpressed in advanced PCa and has been reported to promote disease progression^29^. RNA-seq analyses revealed that SMAD3 knockdown in PCa cells reduced the expression of AR and its target genes, whereas knockdown of SMAD4 or SMAD2 had minimal or no effect^30^. In addition, treatment of PCa cells with TGF-β did not alter AR mRNA levels^30^, suggesting that SMAD3 promotes AR expression independently of TGF-β signaling and the canonical SMAD complex.

ChIP-seq studies identified a prominent SMAD3 binding peak within intron 3 of the AR gene^30^. This peak overlapped with H3K27ac ChIP-seq and assay for transposase-accessible chromatin using sequencing (ATAC-seq) signals in PCa cells and tissues, indicating an active regulatory region. Our unpublished 4C-seq data showed that two independent probes within the SMAD3 peak region physically interact with the AR promoter region. CRISPRi targeting of this SMAD3 peak region resulted in decreased AR mRNA expression^30^, confirming that this region functions as a bona fide enhancer, which we refer to as the AR intron 3 enhancer. Consistently, Hi-ChIP data of H3K27ac in LNCaP cells demonstrated that the AR intron 3 enhancer forms a chromatin loop with the AR promoter^7^.

Analysis of H3K27ac ChIP-seq and ATAC-seq datasets revealed that the AR intron 3 enhancer is activated in PCa tissues^30^. Furthermore, transcriptomic data show elevated levels of both AR and SMAD3 in metastatic PCa and CRPC compared with primary tumors^30^, supporting a role for SMAD3 in driving AR expression in aggressive disease.

Together, these findings suggest that SMAD3 directly binds to the AR intron 3 enhancer to promote AR transcription in advanced PCa. The identification of SMAD3 as a key transcriptional regulator of AR provides a potential therapeutic avenue to inhibit AR signaling. Indeed, treatment with a SMAD3-targeting PROTAC induced SMAD3 degradation and consequently reduced AR expression^30^.

## AR downstream enhancer

A recent study identified the amplification of a potential enhancer element (C4) located approximately 65 kb downstream of the termination site of the AR gene^11^. We refer to this site as the AR downstream enhancer. The copy number of the AR downstream enhancer is increased in CRPC samples after treatment with the AR pathway inhibitors (enzalutamide or abiraterone)^11^. Hi-ChIP data of H3K27ac in LNCaP cells revealed chromatin looping between the AR downstream enhancer and the AR promoter^7^. The motif analysis of ATAC-seq peaks from CRPC organoids suggested the enrichment of transcription factors such as FOXA1 at the AR downstream enhancer region. Consistently, the FOXA1 ChIP-seq peaks overlap with the AR downstream enhancer region in PCa cell lines, primary PCa and CRPC tissues^11^. Future studies are needed to establish whether the AR downstream enhancer functionally contributes to AR expression and CRPC progression.

## Conclusion

Several transcription factors and regulatory proteins have been identified that bind to *cis*-regulatory elements of the AR gene and modulate its expression. Amplification of AR enhancers can further enhance AR transcription. These mechanisms probably contribute to AR upregulation and the development of resistance to androgen deprivation therapy and AR pathway inhibitors in CRPC. Targeting these factors or regulatory elements may offer new strategies for inhibiting AR pathway and improving therapeutic outcomes in advanced PCa.

### Future perspectives

This Review focuses on five *cis*-regulatory elements of the AR gene, including the upstream enhancer, distal promoter, intron 2 enhancer, intron 3 enhancer and downstream enhancer (Fig. 2). H3K27ac ChIP-seq and ATAC-seq data from PCa tissues and organoids reveal that these regulatory regions are co-activated in some datasets but differentially activated in others (Table 1). These observations suggest that these elements may function cooperatively or independently to drive AR expression. Further studies are needed to investigate whether specific regulatory elements are activated at distinct stages of AR-targeted therapy and disease progression, and whether they drive AR overexpression in CRPC independently or cooperatively in a context-dependent manner.

**Table 1:** Activation state of AR *cis*-regulatory elements across PCa datasets. The H3K27ac ChIP-seq and ATAC-seq datasets were downloaded from the GEO database.

GEO number	Tissue type	Method	Sample number	Upstream enhancer	Distal promoter	Intron 2 enhancer	Intron 3 enhancer	Downstream enhancer
GSE161948	PCa-PDX	H3K27ac	22	20	21	17	7	20
		ChIP-seq						
GSE188173	CRPC-PDX	H3K27ac	8	8	8	7	6	8
		ChIP-seq						
GSE193917	PCa organoids	ATAC-seq	22	2	19	6	12	7
GSE193917	PCa-PDX	ATAC-seq	6	5	5	4	4	5

Peaks were visualized using IGV software, and those overlapping with the AR *cis*-regulatory elements in each dataset were identified and counted.

Furthermore, identifying the transcription factors and regulatory proteins that interact with these regions will be critical for understanding how they coordinate AR transcriptional activation. Previous ChIP-seq studies have shown that peaks of key transcription factors such as FOXA1, GATA2 and HOXB13 overlap with multiple *cis*-regulatory regions of the AR gene^5,6,11,31^. Future work should investigate whether additional transcription factors cooperatively regulate AR expression through these *cis*-regulatory elements in CRPC.
